# Four Plasma Glucose and Insulin Responses to a 75 g OGTT in Healthy Young Japanese Women

**DOI:** 10.1155/2018/5742497

**Published:** 2018-01-30

**Authors:** Kei Takahashi, Hidetaka Nakamura, Hiroshi Sato, Hideto Matsuda, Kazuo Takada, Tomiko Tsuji

**Affiliations:** ^1^Department of Health and Nutrition, Faculty of Health and Human Life, Nagoya Bunri University, 365, Maeda, Inazawa-Cho, Inazawa City, Aichi, Japan; ^2^Department of Food and Nutrition, College of Nagoya Bunri University, 2-1, Sasazuka-Cho, Nishi-Ku, Nagoya, Aichi, Japan

## Abstract

The incidence of diabetes has been gradually increasing, not only in middle-aged individuals but also in young individuals. However, insulin and glucose patterns have not been investigated in apparently healthy young individuals, as they are typically grouped as controls. In this study, we investigated and classified glucose and insulin patterns in healthy young women. Sixty-two nonobese women without metabolic disease were recruited. The subjects underwent a 75 g oral glucose tolerance test (OGTT), physical measurements, and a biochemical examination. Two subjects displayed impaired glucose tolerance. The 62 subjects were categorized into four patterns by plasma glucose and insulin peak time during OGTT: normal type (*n* = 39), insulin-late type (*n* = 11), insulin- and glucose-late type (*n* = 7), and insulin-very late type (*n* = 5). OGTT glucose and insulin levels at all time points, insulinogenic index, HOMA-IR, and glucose area under the curve (AUC) significantly differed among the four groups. However, insulin AUC did not significantly differ. We did not detect significant differences in body condition or biochemical measurements. Our study demonstrated that some healthy young individuals might have delayed insulin secretion by OGTT. Early detection of altered glucose metabolism might be helpful to improve lifestyle choices and prevent progression to diabetes.

## 1. Introduction

Chronic diabetes mellitus is characterized by hyperglycemia, which promotes oxidative stress and leads to microangiopathy, such as retinopathy, nephropathy and neuropathy, and macroangiopathy, such as ischemic heart disease and cerebrovascular disease [1, 2]. Recently, it has been reported that not only fasting plasma glucose levels but also postchallenge glucose spikes are problematic in patients with diabetes [3, 4]. For example, cardiovascular disease risk in patients with hyperglycemia 120 min after a meal was higher than in patients with fasting hyperglycemia. Controlling postprandial hyperglycemia is beneficial for prevention and reduction of cardiovascular risks in patients with diabetes [5, 6]. Similarly, patients with type 2 diabetes display delayed early insulin secretion compared with that in healthy adults and are insulin resistant [7]. These reports indicate that postprandial plasma glucose and insulin curves are important.

The incidence of diabetes in younger individuals is gradually increasing [8]. However, there are few studies investigating healthy young subjects. In Japanese universities, most college students do not undergo medical examinations and are not aware of their carbohydrate metabolic conditions. In adults, fasting glucose levels are assessed during a medical examination, but fasting insulin secretion and postchallenge glucose levels are not. The Funagata Study [5], a large Japanese epidemiology study, investigated subjects over 40 years of age but did not include younger individuals. Furthermore, this study did not assess postchallenge insulin levels. Therefore, we believed that it was necessary to assess carbohydrate metabolism and prediabetes status in young healthy individuals.

Mitsui et al. [9] investigated the characteristics of diabetes with isolated fasting hyperglycemia (DM/IFH), with isolated postchallenge hyperglycemia (DM/IPH), and with both fasting and postchallenge hyperglycemia (DM/FPH) in Japanese men. Furthermore, in this study, the DM/IFH and DM/IPH groups were subdivided into two groups and compared. In contrast, young healthy individuals were used as a control group and were not divided into subgroups or analyzed in detail. In several studies investigating diabetes using 75 g oral glucose tolerance tests (OGTTs) [7, 10, 11], healthy individuals were the standard control group with which individuals with diabetes and prediabetes were compared. However, type 2 diabetes in Asia is an increasing epidemic, particularly in individuals at a relatively young age and with a low BMI [8, 12]. Furthermore, Fukushima et al. [13] reported that impaired early-phase insulin secretion played a very important role in the deterioration from normal glucose tolerance to impaired glucose tolerance (IGT) in Japanese middle-aged individuals. Therefore, we thought it necessary to show detailed insulin and glucose patterns in healthy young women during a 75 g OGTT.

In this study, we assessed prediabetes and diabetes by 75 g OGTTs in healthy undergraduates in Japan and investigated varying insulin and plasma glucose responses. In addition, we compared physical and biochemical parameters in the different insulin and glucose response groups.

## 2. Materials and Methods

Ethical approval was obtained from the Research Ethics Committee of the Nagoya Bunri University, and the study was conducted according to the principles expressed in the Declaration of Helsinki. Subjects were informed about the experimental procedures and purpose of the study prior to giving written consent.

Sixty-two female college students were recruited for this study from Nagoya Bunri University in Japan after receiving an explanation of the study. Inclusion criteria for the study were apparently healthy young adults between 19 and 23 years of age with no history of lifestyle-related disease (e.g., diabetes, hypertension, or lipid abnormalities).

The subjects received both physical and blood examinations. Body weight, bone mass, lean body mass, and percent body fat were measured by body composition analyzer InBody 720 (InBody Japan Inc., Tokyo), and body mass index (BMI) was calculated. Subjects underwent a 75 g OGTT according to the recommendations of the World Health Organization [14]. Venous blood samples were obtained directly before (0 min) and during the OGTT (30, 60, and 120 min) for determination of plasma glucose and insulin concentrations. Blood parameters were measured by FALCO Biosystems Ltd. (Kyoto, Japan). Plasma glucose and insulin concentrations were measured by the hexokinase glucose-6-phosphate dehydrogenase method and radioimmunoassay, respectively [15, 16]. Parameters related to protein and lipid metabolism, liver and kidney function, and blood characteristics were measured in blood collected following an overnight fast to confirm the absence of disease. Furthermore, to evaluate carbohydrate metabolism, we calculated homeostasis model assessment-insulin resistance (HOMA-IR), homeostasis model assessment for *β*-cell function (HOMA-*β*) [17], insulinogenic index (II) [18], quantitative insulin sensitivity check index (QUICKI) [19], and area under the curve of glucose (AUC-G) and insulin (AUC-I). HOMA-IR, HOMA-*β*, II, and QUICKI were calculated using the following formulas: insulin_0_ × glucose_0_/405, insulin_0_ × 360/(glucose_0_ − 63), Δinsulin_30_/Δglucose_30_, and 1/[log(insulin_0_) + log(glucose_0_)], respectively, with insulin expressed in *μ*U/mL and glucose in mg/dL. The diagnostic criteria for diabetes from the Japanese Diabetes Society [20] and the World Health Organization [14] were used. The normal type in criteria for diabetes was evaluated at less than 110 mg/dL of fasting plasma glucose levels and 140 mg/dL of 120 min glucose levels. IGT was evaluated at less than 110 mg/dL of fasting plasma glucose levels and from 140 mg/dL to 199 mg/dL of 120 min glucose levels. The diabetes was evaluated with fasting glucose of 126 mg/dL and over or 120 min glucose of 200 mg/dL and over.

Glucose and insulin response types were determined based on the time of peak insulin and plasma glucose concentrations during a 75 g OGTT [21–24], and each parameter was compared. Values are expressed as means ± SD. All data analyses were conducted using Prism (version 5.01; obtained from GraphPad Software, San Diego, CA, USA), and *p* values < 0.05 were considered statistically significant. For the statistical evaluation, the significance of the differences in mean values between the four types were tested by one-way ANOVA followed by Dunn's multiple comparison tests.

## 3. Results

**Figure 1:**
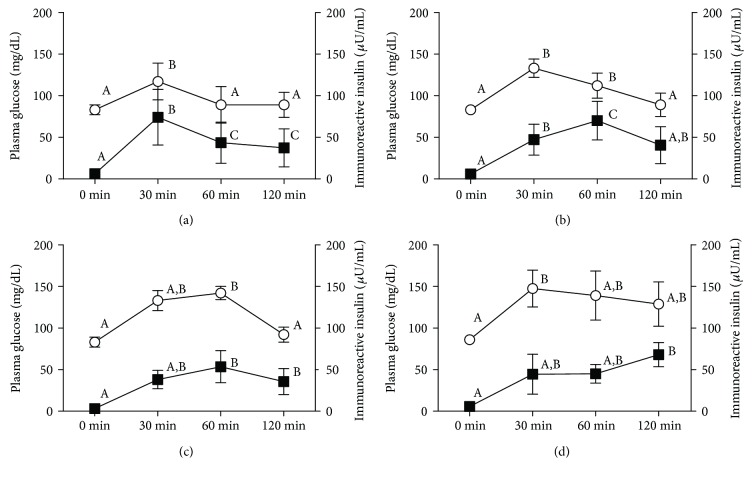
Relationship between plasma glucose levels and insulin reactivity during a 75 g oral glucose tolerance test. Squares: insulin levels. Open circles: plasma glucose levels. (a) Normal type (*n* = 39). (b) Insulin-late type (*n* = 11). (c) Insulin- and glucose-late type (*n* = 7). (d) Insulin-very late type (*n* = 5). Values are means ± SD. Values for each index with different superscript letters are statistically different at *p* < 0.05 by comparing different time points of each patterns, as determined by Dunn's multiple comparison test.

Two of 62 subjects (3.2%) displayed elevated 120 min plasma glucose levels (160 and 187 mg/dL) following oral glucose administration, despite fasting glucose levels below 110 mg/dL, and were, therefore, diagnosed with IGT. This 62 nondiabetic subjects were categorized into four groups based on plasma insulin and glucose peak times, which were the times with the highest insulin and glucose concentrations, respectively, during an OGTT (Figure 1). The four groups were as follows: the peak insulin and glucose times both occurred at 30 min (normal type; *n* = 39 (62.9%), Figure 1(a)); insulin and glucose peak times were 60 min and 30 min, respectively (insulin- (I-) late type; *n* = 11 (17.7%), Figure 1(b)); both peak times occurred at 60 min (insulin- and glucose- (I&G-) late type; *n* = 7 (11.3%), Figure 1(c)); and insulin and glucose peak times were 120 min and 30 min, respectively (I-very late type; *n* = 5 (8.1%), Figure 1(d)). In each group, insulin and plasma glucose concentrations at the peak time were higher than those at baseline (0 min).

Table 1 displays the ages and body conditions based on each OGTT response type. There were no significant between-type differences.

The comparisons of indexes of glucose metabolism in each group are summarized in Table 2. There were no significant differences in plasma glucose levels at 0 min among the four groups. The 60 min plasma glucose concentrations in the I-late and I&G-late type groups were significantly higher than those in the normal type group, but there were no differences in 120 min glucose levels. In I-very late type group, the 30 min, 60 min, and 120 min glucose levels were significantly higher than those in normal type. In contrast, 0 min and 30 min immunoreactive insulin levels in the I&G-late type group were lower than those in the normal and I-late type groups. The 60 min insulin concentrations in the I-late type group were higher than those in the normal type group. And the 120 min insulin in I-very late group was higher than those in the normal. HOMA-IR and HOMA-*β* in the I&G-late type group was lower while QUICKI values were higher than those in the other groups. II values in the normal type group was higher than those in the other type groups. AUC-G in the I&G-late and I-very late type groups were higher than that in the normal type group, but there were no significant differences in AUC-I among the four groups.

Additional biochemical parameters are displayed in Table 3. There were no significant differences in any parameter among types.

## 4. Discussion

This study was performed in young healthy women without lifestyle-related diseases or other diseases. They underwent a 75 g OGTT and were categorized in four groups based on peak times of plasma insulin and glucose. There were several studies categorized with insulin secretion time. Hayakawa [21] and Kai et al. [22] divided the two insulin types into type with peak time from 30 min to 60 min (normal) and those with peaks at 120 min. Sasaki et al. [23] and Tanabe et al. [24] divided the three insulin types into type with peak time at 30 min, 60 min, and 120 min, and we used this classification in this study. However, they divided the types without considering the peak time of plasma glucose levels. In this study, we divided the types considering not only insulin peak time but also plasma glucose peak, and as a result we could divide them into four types. There were no subjects with fever or menstruation on the day of the experiment. Even if there is a missing declaration, insulin secretion is more susceptible to pregnancy and aging than menstruation [25], so we think that it does not have a significant influence on grouping of subjects.

In this study, we were unable to investigate physical activity or food requirements. However, our subjects were studying nutritional science to become dietitians and might, therefore, have better exercise and dietary habits than general college students. The number of subjects in each group were not equal, and the statistical difference might be hard to confirm, especially for the I-very late type group. However, it was important to reveal patterns prior to the appearance of a carbohydrate metabolic disorder in young healthy women, because it might facilitate diabetes prevention.

We did not identify subjects with abnormal fasting plasma glucose levels or other blood characteristics but did identify two subjects with IGT and four distinct carbohydrate patterns in healthy subjects by OGTT. In the two subjects with IGT, BMIs were below 25 kg/m^2^ and the insulin peak time was 120 min after glucose administration. The other biochemical parameters were normal, and they did not have a family medical history of diabetes in a relative within the second degree of relationship.

Relative to the normal group, insulin secretion was delayed in the I-late and I&G-late groups and even further delayed in the I-very late group. And the number of subjects with normal response patterns was greater while that with abnormal patterns was smaller. However, this might be because this study was conducted only with young healthy women. If we did it with a larger number of subjects including men middle aged and older, this ratio might have changed. In the normal type group (Figure 1(a)), insulin secretion increased until 30 min after glucose administration, at which time, plasma glucose and insulin levels gradually decreased. Therefore, only glucose values at 30 min, which also corresponded to the insulin peak time, were significantly higher than 0 min glucose values. Glucose levels at 60 and 120 min declined, returning to concentrations comparable to those at baseline. Compared with that of the normal group, the I-late and I&G-late type groups displayed intermediate impairment of carbohydrate metabolism. The subjects in these groups displayed delayed insulin secretion following glucose administration. In the I-late type group (Figure 1(b)), in which the peak time for insulin was 60 min and glucose was 30 min, insulin sensitivity might be normal, because the increase in insulin secretion from 30 to 60 min resulted in a decrease in glucose levels. In addition, insulin secretory capacity was normal, because HOMA-IR, HOMA-*β*, AUC-G, and AUC-I, which are indices of carbohydrate metabolism, were not significantly different from the normal type group (Table 2). The third group was the I&G-late type group (Figure 1(c)), characterized by peak insulin and glucose times at 60 min. This group displayed not only an insulin secretory delay but also a secretion shortage during fasting and the early stages of the OGTT. For example, immunoreactive insulin levels and HOMA-IR values, which were calculated using fasting glucose and insulin levels, were significantly lower than those in the control and I-late type groups (Table 2). II values, which were calculated using glucose and insulin levels at fasting and 30 min after glucose administration, were lower than those in the normal groups, because early insulin secretion after glucose administration was the lowest. However, although the 60 min insulin level was the peak value in the I&G-late group, it did not differ significantly from that in the normal type group. AUC-I values in the I&G-late type group did not differ from the other groups. These results suggest that fasting and early insulin secretion were lower than those in the normal group, but total secretion was comparable. The final group was the I-very late type group, characterized by a peak insulin time at 120 min and peak glucose time at 30 min. This insulin and glucose pattern was similar to subjects with IGT. Insulin secretion during the early stage was low, and glucose levels remained elevated above 110 mg/dL at 120 min post-glucose administration, despite maintained insulin secretion between 30 and 120 min (Table 2 and Figure 1(d)). This phenomenon was caused by a secretion delay and not insulin resistance, because HOMA-IR was not abnormal and AUC-I values did not significantly differ from those in the normal type group. Tanabe et al.'s study [24] in healthy people averaging 60 years old showed that the insulin peak at 120 min was 25 out of 680 (3.7%). Though we had 3 people (5.0%) in our I-very late type group without IGT subjects, it was more than that proportion. This phenomenon might be consistent with the gradually increasing number of young individuals with diabetes [8].

In this study, we did not detect differences in AUC-I levels between the four groups but did find differences in AUC-G levels and peak times. This result suggests that differences in carbohydrate metabolism in healthy young women were caused by different insulin secretion times and not secretory capacity or insulin resistance. However, the mechanisms underlying differences in secretion times remain unclear. For example, these differences might be caused by genetic background, obesity, exercise habits, skipping breakfast, having a midnight meal, and speed eating [12, 26–29]. However, in this study, there were subjects with BMIs above 25 kg/m^2^ in the normal and I-late type groups but not in the I&G-late and I-very late type groups. In each group, there were subjects with a family medical history of diabetes in a relative within the second degree of relationship, and this did not differ between groups (data not shown). Biochemical parameters, including HOMA-IR and II, were normal. We did not investigate the other factors in this study. Kikuoka et al. demonstrated that 13.1% of subjects with impaired fasting glucose progressed to type 2 diabetes within 5 years [30]. Future studies will investigate lifestyle habits and conduct a follow-up survey of the four insulin and plasma glucose response groups.

Normal indices of carbohydrate metabolism (e.g., fasting plasma glucose, HOMA-IR, and II) did not allow us to classify the subjects into four groups. For example, if we classified only with peak insulin times, we might not have noticed the significant differences in HOMA-IR, QUICKI, and immunoreactive insulin levels between the I-late type and I&G-late type groups. If we classified subjects only according to peak glucose times, we might not have noticed the maintenance of hyperglycemia between 30 and 120 min in the I-very late type group. We revealed these differences by using both peak glucose and insulin times. In our study, about 37% of subjects had delayed insulin secretion. In a typical study, these individuals might be categorized into the control group. In our 60 subjects, excluding two IGT subjects, the average plasma glucose levels were 83.6 ± 5.6, 123.3 ± 21.3, 100.8 ± 27.0, and 90.8 ± 15.2 mg/dL at 0, 30, 60, and 120 min post-glucose administration, respectively. The average insulin levels were 5.8 ± 2.5, 68.1 ± 42.4, 52.4 ± 37.5, and 41.6 ± 30.8 *μ*U/mL, respectively. These values were similar to normal type values in this study, and thus, individuals with delayed insulin secretion, who might be at risk for diabetes, would have been overlooked in the healthy control group. We believe that an understanding of one's own carbohydrate metabolism is important for type 2 diabetes prevention. OGTTs may allow us to assess glucose and insulin response types.

## 5. Conclusions

We revealed that insulin secretion and plasma glucose patterns could be categorized into four groups, despite all subjects being healthy young women, because postchallenge and fasting insulin secretion varied among individuals. The first group was the normal type group, in which insulin and glucose levels 30 min after glucose administration during an OGTT were highest and then gradually decreased. The second type displayed delayed postchallenge insulin secretion. However, other indices of carbohydrate metabolism (e.g., HOMA-IR, HOMA-*β*, AUC-I, and AUC-G) were not significantly different. The third group displayed delayed and reduced insulin secretion. In this group, fasting insulin levels were low, and the lack of additional insulin secretion caused a delay in peak plasma glucose levels. The fourth group displayed very late postchallenge insulin secretion, and this group contained IGT subjects. The delayed secretion caused persistent postprandial hyperglycemia until 120 min after glucose administration. Even in some healthy young women, insulin secretion was delayed after oral glucose administration, and they might, therefore, need improvements in their lifestyle habits. Confirmation of insulin secretion and plasma glucose patterns in young healthy individuals might be helpful for early type 2 diabetes prevention. Future studies should compare lifestyle habits among the groups and conduct a follow-up survey to identify subjects that develop IGT.

## Figures and Tables

**Table 1:** Body condition of healthy women by insulin and glucose response type.

Type	Normal	Insulin-late	Insulin- & glucose-late	Insulin-very late
Number	39 (62.9%)	11 (17.7%)	7 (11.3%)	5 (8.1%)
Age (years)	20.6 ± 0.9	20.4 ± 1.0	19.9 ± 1.1	20.7 ± 0.5
Body weight (kg)	52.3 ± 6.9	53.1 ± 6.8	52.1 ± 6.2	53.3 ± 2.2
Height (cm)	158.0 ± 4.5	157.7 ± 5.1	160.9 ± 8.1	158.3 ± 1.7
Body mass index (kg/m^2^)	20.9 ± 2.3	21.4 ± 1.0	20.1 ± 1.4	21.3 ± 1.0
Bone mass (kg)	2.23 ± 0.21	2.25 ± 0.11	2.18 ± 0.31	2.23 ± 0.11
Lean body mass (kg)	37.2 ± 3.3	37.8 ± 1.9	36.4 ± 5.6	38.2 ± 1.6
Percent body fat (%)	27.0 ± 4.8	27.5 ± 4.5	28.6 ± 4.7	29.6 ± 3.7

Values are means ± SD. There were no significant differences.

**Table 2:** Glucose metabolism of women by insulin and glucose response type.

Type	Normal	Insulin-late	Insulin- & glucose-late	Insulin-very late
Number	39	11	7	5
Plasma glucose (mg/dL)				
0 min	83.4 ± 6.1	83.5 ± 4.5	83.0 ± 5.7	86.0 ± 3.0
30 min	117.0 ± 22.2^a^	133.2 ± 10.9^a,b^	133.1 ± 12.0^a,b^	147.4 ± 22.1^b^
60 min	89.2 ± 22.2^a^	112.1 ± 14.5^b^	142.0 ± 15.6^b^	139.0 ± 29.4^b^
120 min	89.3 ± 14.8^a^	88.8 ± 13.5^a,b^	92.0 ± 9.4^a,b^	114.7 ± 24.8^b^
Immunoreactive insulin (*μ*U/mL)				
0 min	6.2 ± 2.7^a^	5.7 ± 1.9^a^	3.2 ± 0.8^b^	5.7 ± 1.4^a,b^
30 min	74.1 ± 33.4^a^	47.2 ± 18.6^a^	38.1 ± 11.1^b^	44.5 ± 24.0^a,b^
60 min	42.3 ± 23.7^a^	65.9 ± 26.0^b^	53.5 ± 19.2^a,b^	45.0 ± 12.9^a,b^
120 min	36.1 ± 21.5^a^	40.6 ± 22.1^a,b^	35.6 ± 15.6^a,b^	68.0 ± 14.5^b^
HbA1c (%)	5.18 ± 0.27	5.12 ± 0.22	5.05 ± 0.20	5.10 ± 0.17
HOMA-IR	1.32 ± 0.63^a^	1.23 ± 0.43^a^	0.66 ± 0.18^b^	1.20 ± 0.26^a,b^
Insulinogenic index	2.50 ± 2.14^a^	1.10 ± 0.94^b^	0.76 ± 0.38^b^	0.59 ± 0.25^b^
HOMA-*β*	115 ± 47^a^	108 ± 44^a,b^	65 ± 33^b^	92 ± 35^a,b^
QUICKI	0.38 ± 0.03^a^	0.38 ± 0.03^a^	0.42 ± 0.02^b^	0.38 ± 0.02^a,b^
AUC-glucose (hr·mg/dL)	191 ± 31^a^	216 ± 21^a,b^	240 ± 11^b^	253 ± 53^b^
AUC-insulin (hr.*μ*U/mL)	91.9 ± 44.8	94.7 ± 38.5	77.8 ± 27.3	88.0 ± 29.8

Values are means ± SD. HOMA-IR: homeostasis model assessment-insulin resistance; HOMA-*β*: homeostasis model assessment for *β*-cell function; QUICKI: quantitative insulin sensitivity check index; AUC: area under the curve. The values for each index with different superscript letters are statistically different at *p* < 0.05 by comparing different patterns of each time, as determined by Dunn's multiple comparison test.

**Table 3:** Biochemical status of woman by insulin and glucose response type.

Type	Normal	Insulin-late	Insulin- & glucose-late	Insulin-very late
Number	39	11	7	5
Total protein (g/dL)	7.67 ± 0.40	7.50 ± 0.45	7.28 ± 0.25	7.30 ± 0.43
Albumin (g/dL)	4.70 ± 0.25	4.79 ± 0.16	4.80 ± 0.22	4.63 ± 0.35
Albumin/globulin ratio	1.73 ± 0.25	1.84 ± 0.36	2.00 ± 0.32	1.75 ± 0.21
AST (U/L)	17.3 ± 3.2	17.6 ± 3.7	20.2 ± 6.1	15.5 ± 2.4
ALT (U/L)	13.3 ± 4.9	12.6 ± 5.8	14.8 ± 8.1	14.0 ± 6.8
*γ*-GTP (U/L)	17.7 ± 8.8	16.1 ± 4.9	16.2 ± 4.4	20.0 ± 8.4
LDL-C (mg/dL)	91.7 ± 21.3	90.0 ± 19.8	86.7 ± 13.8	105.3 ± 19.4
Triglyceride (mg/dL)	57.8 ± 22.7	43.0 ± 9.8	48.2 ± 16.1	68.5 ± 48.8
HDL-C (mg/dL)	68.9 ± 14.9	76.2 ± 7.8	73.3 ± 14.9	66.8 ± 13.7
Blood uric nitrogen (mg/dL)	11.7 ± 2.5	12.3 ± 4.5	10.3 ± 2.1	16.0 ± 6.7
Serum creatinine (mg/dL)	0.57 ± 0.08	0.58 ± 0.05	0.59 ± 0.08	0.55 ± 0.08
Serum uric acid (mg/dL)	4.18 ± 0.76	4.17 ± 0.74	4.18 ± 1.05	4.98 ± 0.87

White blood cell count (×10^2^/*μ*L)	60.7 ± 15.5	57.5 ± 11.7	55.1 ± 11.6	60.0 ± 14.3
Red blood cell count (×10^4^/*μ*L)	446 ± 28	451 ± 36	459 ± 27	444 ± 19
Hemoglobin (g/dL)	13.3 ± 0.9	13.3 ± 1.3	12.6 ± 2.7	13.3 ± 0.9
Hematocrit (%)	40.9 ± 2.2	40.1 ± 3.7	40.3 ± 6.5	40.6 ± 2.3
MCV (fL)	91.9 ± 3.5	90.6 ± 4.0	87.4 ± 11.4	91.4 ± 3.4
MCH (pg)	29.8 ± 1.8	29.4 ± 1.7	27.3 ± 5.3	30.0 ± 1.4
MCHC (%)	32.4 ± 1.2	32.5 ± 0.9	30.9 ± 2.7	32.8 ± 1.1
Platelet count (×10^4^/*μ*L)	26.6 ± 5.1	26.7 ± 6.0	24.7 ± 8.0	26.4 ± 4.2

Values are means ± SD. There were no significant differences.
